# How do ADHD children perceive their cognitive, affective, and behavioral aspects of anger expression in school setting?

**DOI:** 10.1186/1753-2000-4-4

**Published:** 2010-01-25

**Authors:** Ahmad Ghanizadeh, Habib Bagherpour Haghighi

**Affiliations:** 1 Research Center for Psychiatry and Behavioral Sciences, Shiraz University of Medical Sciences, Hafez Hospital, Shiraz, Iran; 2 Shiraz University of Medical Sciences, Shiraz, Iran

## Abstract

**Background:**

Anger is an ignored research area in children and young adolescents with Attention deficit hyperactivity disorder (ADHD) in the school setting. This study compares school anger dimensions in children and young adolescents with ADHD and a control group.

**Methods:**

The subjects were a clinical sample of 67 children and young adolescents with ADHD and their parents, with a sample of 91 children from the community of similar age and gender as control group. Anger was measured by the Farsi version of the Multidimensional School Anger Inventory (MSAI).

**Results:**

The scores of the two components of "Hostile Outlook" and "Positive Coping" were different between the groups. The mean scores for the Anger components did not statistically differ between the children with ADHD and ODD and ADHD without ODD, boys and girls, or different types of ADHD.

**Conclusion:**

Children with ADHD do not report higher rates of experience of anger and they do not apply destructive strategies more than the control group. However, children with ADHD appear to have a more hostile outlook toward school and their coping strategy is weaker than that of the control group.

## Introduction

Attention deficit hyperactivity disorder (ADHD) is one of the most common psychiatric disorders in children and adolescents[1]. The core symptoms of ADHD are inattentiveness, hyperactivity, and impulsivity [2]. Emotional dysregulation has been reported to be an important component of ADHD [3]. One of the most common reasons for the clinical referral of children and adolescents to child psychiatry clinics is aggression. Recently, population-based studies on schoolchildren have demonstrated a connection between ADHD and bullying, a specific form of aggressive behavior [3,4]. Meanwhile, both of the parents' and teachers' knowledge about ADHD is not high [5,6] and disclosure of ADHD label is not associated with rejection of students by their teachers [7].

Anger is a state of arousal resulting from social conditions involving threat or frustration. It is a strong feeling of displeasure that includes a sense of antagonism. Anger expression is not the equivalent construct of aggression. Aggression consists of verbal and/or physical acts aimed at hurting or upsetting another human being either directly or indirectly. Aggression is a behavior that may be associated with anger [3]. Anger is an unpleasant experience for the angry person and those who are involved. The negative outcomes of anger are not limited to aggression and violence [8]; there are many other negative outcomes such as interpersonal relationships problem, school problems, anxiety and depression [9,10], drug abuse [11], and even health problems such as hypertension [12] cardiovascular disease and psychosomatic symptoms [13,14].

Anger expressed by aggression may lead to punishment of the student, such as being removed from the class or even expulsion from the school. Aggression in young people has become a major concern and self-reported aggressive and violent behaviors have risen among children. In one self-report study, 54.3% of the students reported committing at least one act of violence in the current academic year; and about one third of the students reported high levels of anger-expression [15]. Furthermore, the mean effect size for school-based interventions related to reducing anger is 0.31, which is considered very low [16]. This low effectiveness of anger-related interventions was related to the co-occurrence of anger with some other problems such as attention-related symptoms, which are important in the management of anger [16]. The effect size of stimulants on overt and covert aggression-related behavior in ADHD children are 0.84 and 0.69, respectively. However, co-morbid problems decreased the effect sizes [17].

In the light of these serious unfavorable outcomes of anger, studying anger in students is very important. The influence of anger is largely unexplored and has received less attention probably because it is difficult to quantify or discretely categorize anger. Anger can be assessed by self-reports from about seven years old onwards [18]. These children are able to properly discriminate this emotion if questions are formulated in short and plain sentences [18]

Gender differences do not appear to be significant in terms of anger [13]. Children of either gender with high anger levels have poor cognitive processing skills and poor relationships in school [19]. Children with a diagnosis of ADHD demonstrate aggressive behavior and have difficulties for interpreting social codes more often than their peers [4]. On the other hand, a study on boys with ADHD in an aggressive game showed that manipulation of their background anger did not affect their aggressive behavior [20]. A self-report questionnaire study of 23 boys diagnosed with ADHD and without co-morbid oppositional defiant disorder (ODD) or conduct disorder suggests that the children with ADHD negotiate their angry feelings towards a friend with him/her less often than those in a control group. Boys with ADHD report anger regulation strategies that require impulse control less often than their counterparts without ADHD [21].

This current study compares school anger dimensions in ADHD children and a control group of children from the community of a similar age range. It also surveys the impact of gender and co-morbidity of ODD on school anger dimensions in ADHD children. It was hypothesized that children with ADHD experience more anger and use more destructive expressions of anger than a control group of their peers. To the best of the authors' knowledge, there is no prior study exploring the relationship between anger and ADHD in children and younger adolescents as young as those in the current study.

## Methods

The study sample consisted of 158 children: 67 children and adolescents diagnosed as having ADHD and their parents, and 91 children from general population of similar age and gender.

ADHD was diagnosed according to Diagnostic and Statistical Manual of Mental Disorders, Text Revised criteria using [2]. The children and adolescents with ADHD were consecutive referrals of the outpatient clinic of Hafez Hospital affiliated to the Department of Child and Adolescent Psychiatry at Shiraz University of Medical Sciences. All the 67 children and adolescents (with an age range of 8 to 14) and their parents were interviewed by the child and adolescent psychiatrist who separately interviewed the child and the parents, administering the Schedule for Affective Disorders and Schizophrenia for School Age Children (K-SADS), Farsi version [22]. The Farsi version of the K-SADS is a semi-structured psychiatric interview with satisfactory reliability and validity [22]. There is also sufficient test-retest and inter-rater reliability [22]. Test-retest reliability of ADHD is 0.81. Inter-rater reliability of ADHD is 0.69 [22]. The K-SADS covers other pediatric psychiatric disorders such as oppositional defiant disorder (ODD).

The control group consisted of 91 schoolchildren from Shiraz, Southern Iran. The demographic data of the control group were collected using a self-administered questionnaire which asked for children's age, gender, and the rank of birth. The age range was nine to fifteen years, with the school classes surveyed being selected to correspond to the age range of the sample group. The trained evaluator distributed the questionnaires among the middle school students. He briefly explained the study objectives and provided the necessary instructions. The students then completed the questionnaires during the class time. The questionnaires were anonymous and taking part in the study was voluntary. The response rate of the sample was about 85%.

Anger was measured by the Farsi version of the Multidimensional School Anger Inventory (MSAI). MSAI is a self-report questionnaire that measures anger components within a school context [23]. Its components are affective, cognitive, and behavioral dimensions of anger specific to the general school setting and context, conceptualized as the experience of anger, hostility of outlook, and the expression of anger respectively (See Table 1). Each of the components measures a relatively independent aspect of the general construct of anger. The MSAI is a reliable and valid measure of anger experience and expression and it is relatively independent of aggression [24].

**Table 1:** Comparison of the mean score of anger components among subtypes of ADHD

ADHD subtypes (Total sample size = 67)	School Anger Experience Scale mean score (SD)	Hostile Outlook mean score (SD)	Positive coping mean score (SD)	Destructive Expression mean score (SD)
Inattentive (n = 24)	34.7(7.6)	9.2(3.4)	10.7(3.4)	10.0(1.9)
Combined (n = 29)	30.1(8.6)	10.3(4.1)	11.2(3.7)	11.0(3.0)
Hyperactive/impulsive (n = 14)	35.5(6.9)	8.5(2.4)	10.9(3.)	11.3(2.6)
Means t-test P Value	.061	.432	.897	.341

The Farsi version of MSAI has 28 items, each with a four-point Likert-type response format. There are four different categories with their own response type: the School Anger Experience Index, the Hostile Outlook, the School Anger Expression Index (Positive Coping) and the School Anger Expression Index (Destructive Expression). The 12 Anger Experience Index items have the following responses: one (I'm not angry at all), two (I'm a little bit angry), three(I'm pretty angry), and four (I'm very angry. I'm furious). This category assesses anger experience with hypothetical but common school situations in which the students report their own real or likely level of anger. The range of possible scores on this component is 12 to 48. Examples of the questionnaire items within School Anger Experience are: "You tell the teacher that you are not feeling well but he/she doesn't believe you", "You ask to go to the bathroom and the teacher says no", and "Somebody calls you a bad name". The responses of the six Hostile Outlook items are: one (strongly disagrees), two (disagree), three (agree), and four (strongly agree). The range of possible scores on this component is 6 to 24. This component assesses the students' level of school hostility to a series of statements. Some of the items of this component are: "School is really boring", "Grades at school are unfair", and "Rules at school are stupid". The responses of the 10 items of School Anger Expression component are: one (never), two (occasionally), three (often), and four (always). This component represents positive or destructive coping strategies in which the students reported how often they may use them in dealing with their anger. The range of possible scores on both Positive Coping of School Anger Expression Index and Destructive Expression for School Anger Expression Index categories is 5 to 20. Destructive expression includes aggressive responses and positive coping includes more socially acceptable responses. Some of the items of Positive Coping of School Anger Expression Index are: "When I'm upset, I calm myself down by reading, writing, painting, or some similar activity" and "Before I explode, I try to understand why this happened to me". Some of the anger Expression Index (Destructive Expression) items are: "When I'm mad, I break things", "I get so mad that I want to hurt myself", and "If I get mad, I'll throw a tantrum". Higher scores in the components of School Anger Experience, Hostile Outlook and Anger Expression Index (Destructive Expression) suggest high levels of anger coupled with poor coping mechanisms with anger. Conversely, higher scores in the positive coping category suggest better and more pro-social coping mechanisms with respect to anger.

The Farsi version of MSAI has acceptable validity and reliability. The internal consistency reliability for the questionnaire categories are as follows: of Anger Expression 0.89, of Hostile Outlook 0.79, of Positive Coping Anger Expression 0.66 and of Destructive Expression 0.71. The reliability of the questionnaire as a whole is 0.81 [25].

The protocol for the research project conforms to the provisions of the Declaration of Helsinki in 1995 as revised in Edinburgh 2000. All of the patients and their parents agreed to participate in the study and gave informed consent.

### Analysis

Chi-square tests and means t-tests were used to compare demographic characteristic of the two groups where they were applicable. The mean differences of scores on the categories of the MSAI between the ADHD children and control group were compared using t-tests. Mann-Whitney Tests were used to compare the mean differences of questionnaire categories between ADHD children co-morbid with ODD and those ADHD children without ODD co-morbidity. The Kruskal-Wallis Test was used to compare the mean score of the different questionnaire categories among subtypes of ADHD.

## Results

Demographic characteristics of the ADHD children, the control group, and their parents are displayed in Table 2. Fifty-three children (79.1% of 67) of the ADHD group and sixty-seven children (73.6% of 91) of the control group were boys. There was no statistically significant difference between the two groups regarding the proportion of boys (χ^2 ^= 0.63, df = 1, P = .4). The age range of the ADHD children group was 8 to 14 years and 9 to 15 years for the control group (exact ages in months and years were not collected). The mean (SD) age of the ADHD children and control group was 10.6(1.4) and 11.7(1.8) years, respectively, with no significant difference between the two groups (Table 2). The two groups were not different in terms of rank of birth of the children (χ^2 ^= 2.7, df = 3, P = 0.4). Amongst the children in the ADHD sample group, the breakdown of ADHD subtypes were 24(35.8%), 29(43.3%), 14(20.9) for Inattentive, Combined, and Hyperactive-Impulsive types, respectively (Table 1) The mean score difference of the four categories of the MSAI were not statistically significant between subtypes of ADHD (Table 1). The rate of co-morbidity with ODD in the ADHD children was 56.7%.

**Table 2:** Demographic characteristics of the children and their parents

		ADHD group Mean (SD)	Community group Mean (SD)	Significance
Children	Age in years	10.6(1.4)	11.07(1.8)	t = 1.52, df = 156, P = 0.1
	Number of boys (%)	5373.6%)	61(79.1%)	χ^2 ^= 0.63, df = 1, P = 0.4
	Number of siblings	1.7(1.1)	2.6(1.3)	t = 4.2, df = 156, P < 0.001
Father	Age in years	42.5(6.8)	41.2(6.3)	t = 1.2, df = 156, P = 0.2
	Years of Educational level	9.3(5.3)	10.3(3.5)	t = 1.4, df = 156, P = 0.1
	Number of employed (%)	49(96.1%)	78(94.0%)	χ^2 ^= 0.02, df = 1, P = 0.5
Mother	Age in years	35.2(6.0)	35.1(5.8)	t = 0.14, df = 145, P = 0.8
	Years of Educational level	10.(3.9)	9.4(3.6)	t = 2.03, df = 156, P < 0.04
	Number of housewives (%)	84(82.3%)	51(93.3%)	χ^2 ^= 4.5, df = 1, P < 0.03

Table 3 shows that the scores for two out of the four questionnaire categories, those of Hostile Outlook and School Anger Expression Index (Positive Coping), were significantly different between the ADHD and control groups. The effect sizes were 0.43 and 0.39, respectively. The ADHD children's attitudes towards school were more hostile than the control group, and children with ADHD had less positive coping strategies than the control group.

**Table 3:** Comparison of mean score of the Anger Components between the children with ADHD and the control group

Anger Category of MSAI	Group		Significance
		
	ADHD Mean score (SD)	Control Mean score (SD)	
**School Anger Experience Index**	32.9(8.2)	34.8(8.0)	t = 1.5, df = 156, P = 0.1
**Hostile Outlook**	9.5(3.6)	8.2(2.3)	t = 2.8, df = 156, P < 0.005
**School Anger Expression Index (Positive Coping)**	11.0(3.5)	12.3(3.1)	t = 2.4, df = 156, P < 0.01
**School Anger Expression Index (Destructive Expression)**	10.7(2.6)	10.6(3.0)	t = .30, df = 156, P = 0.7

Comparisons of the mean scores of the questionnaire categories between the ADHD children with and without ODD are presented in Table 4. None of the scores in the questionnaire categories was different between the two groups. Table 5 presents the gender differences of anger components in children with ADHD. Experience or expression of anger and positive coping strategies scores were not different between boys and girls.

**Table 4:** Comparison of mean score of the anger components between the ADHD children with and without ODD)

Anger category of MSAI	ADHD children		Significance
		
	With ODD (n = 40) Mean score (SD)	Without ODD (n = 27) Mean score (SD)	
School Anger Experience Index	32.4(8.3)	33.6(8.2)	P = 0.52
Hostile Outlook	10.0(4.1)	8.8(2.6)	P = 0.35
School Anger Expression Index (Positive Coping)	10.9(3.6)	11.1(3.4)	P = 0.80
School Anger Expression Index (Destructive Expression)	11.0(2.7)	10.2(2.4)	P = 0.15

**Table 5:** Difference between ADHD boys and girls in the anger components

Anger category of MSAI	Gender	N	Mean (SD)	**Sig**.
School Anger Experience Scale	girl	14	32.4 (7.2)	0.7
	boy	53	33.0(8.5)	
Hostile Outlook	girl	14	10.0(3.8)	0.4
	boy	53	9.4(3.5)	
Positive Coping	girl	14	12.5(3.8)	0.07
	boy	53	10.6((3.4)	
Destructive Expression	girl	14	11(2.4).	0.1
	boy	53	10.5(2.6)	

## Discussion

The aim of this study was to compare the school anger components between children and young adolescents with ADHD and a population control group of their peers. The main symptoms of ADHD are impulsivity and difficulty in focusing of attention, and children and adolescents with ADHD are often impaired and frustrated in both academic achievement and social relationships as a consequence. Therefore, it was hypothesized that the School Anger Experience Index score, Hostile Outlook score and School Anger Expression Index (Destructive Expression) scores on the MSAI of children with ADHD would be higher than those of the control group, with the School Anger Expression Index (Positive Coping) scores being correspondingly lower. We did not find that the School Anger Experience Index scores or the School Anger Expression Index (Destructive Expression) scores of children with ADHD were different from those of the control group. However, the Hostile Outlook scores were higher and the School Anger Expression Index (Positive Coping) scores were lower in children and young adolescents than that of the control group. This suggests that children with ADHD may not experience anger or apply destructive and harmful anger strategies more than the general population within the school context. However, the results also suggest that children with ADHD may re-appraise angry situations or try to get away from these situations less frequently than their peers. Further, the results suggest that their ability to take a cognitive approach to resolving anger may also be weaker. We hypothesize that the attitude of children with ADHD toward school may be more negative and that they may interpret situations in the school context as providing more cause for anger, more frequently. These hypotheses are consistent with results from a similar previous study which studied anger in an older group of young people with ADHD [21].

It should be noted that the participants with ADHD in the current study are relatively young and newly diagnosed. This group may not as yet have experienced the longer-term negative effects of ADHD or adopted negative attitudes as a result of consequent difficulties in school and peer relationships. As children and young adolescents with ADHD move into older adolescence and adulthood, they face different negative outcomes and consequences of their symptoms and these may lead to the development of higher levels of anger and hostility than we have found in this study. This therefore does not rule out the possibility that children and young adolescents with newly-diagnosed ADHD who have relatively few problems with anger in the school context may experience more anger and engage in more destructive expression as they grow older. Alternatively, children may learn to repress their anger more extensively with increasing age because they no longer believe that they should express negative emotions as freely [26]. Moreover, research suggests that younger children are more likely get outwardly angry with their parents than with peers [26], and that the expression of anger may vary according to the relational context in which it takes place [24]. A questionnaire study looking at anger and hostility in the school context may not find differences between children with ADHD and their peers in other social contexts, in particular within the family.

The MSAI Hostile Outlook scores of children with ADHD were significantly higher than that of the control group. This finding suggests that the children with ADHD have a more hostile outlook towards school. Some of the items of this component are "School is worthless" and "School is really boring". This finding of higher levels of hostility towards school is consistent with a study of a clinical sample of children with school refusal which reported that ADHD was one of the commonest co-morbid psychiatric disorders in this group [27].

The lack of association between anger components and different types of ADHD found in this study does not tend to support any hypothesis that ADHD children of the hyperactive-impulsive type experience and express more anger than the other subtypes. However, the numbers in each subgroup were low and the study probably did not have sufficient power to demonstrate any differences between subgroups. This study did not find that boys and girls with ADHD had any differences in their scores in the categories of the MSAI. This is inconsistent with other research which suggests that girls generally display less anger than boys [28]. This failure to find any gender difference may be a Type II error occurring because of the low number of girls with ADHD in this study.

We did not find any differences between scores in the anger expression categories of the ADHD children with and without ODD. Children usually display their anger more extensively in specific settings such as their homes. It is possible that children with ADHD, both with and without ODD, feel unsafe to express anger in the school setting; or that they have learned not to share those feelings in school.

There are some limitations to this study. This was a cross-sectional study of self-perceived anger with a single informant, which means that recall bias and minimization of negative emotions and behaviour may limit its generalization. It is known that children with ADHD underreport their symptoms [29], which suggests that they may also underreport emotional, behavioural and social difficulties. As the primary aim of the study was not comparing anger expression among ADHD subtypes, lack of difference between the ADHD types and between boys and girls may be due to type II error. A previous study on girls with ADHD reported that those with ADHD-C exhibited higher rates of overt and relational aggression than those with ADHD-I [30]. The age range of participants is both chronologically and developmentally broad, and analyses within tighter age ranges are needed in order to ensure that age-related increases in emotionality do not influence the results. Also, further studies should account for the co-occurrence of psychiatric disorders other than ODD in order to better interpret the results. Anger relating to school setting cannot be generalized to global experiences of the feeling of anger. Future research should investigate whether results are similar across older age groups and other contexts. The study of anger in ADHD could be strengthened with additional, more objective, measures such as interview methodologies for anger assessment or behavioral observations by trained observers. The current findings were from a clinical sample of ADHD children. This study has used a clinical sample and should be replicated in children with ADHD within the community. The control group was not interviewed to assess for the presence of ADHD or other psychiatric disorders, and given the prevalence of psychiatric disorders, it is likely that a few children within the control group would be suffering from undiagnosed mental health problems. This may reduce power of the current study to detect any differences. This study, with its cross-sectional design, can only look for correlations and does not allow any imputation of a causal relationships between any component of anger and ADHD. The instrument used to assess anger components is not comprehensive. Other dimensions of anger expression such as anger control and the effects on targets of anger such as classmates, teachers, parents, and siblings should be studied in further studies.

## Conclusion

Children and young adolescents diagnosed with ADHD report more hostility towards school and a lower positive coping strategy for controlling anger in school. The results suggest that an awareness of this hostility towards school and less positive coping in children and young adolescents with ADHD may be clinically useful as these could be promising targets for school-based early interventions. School nurses and educational and clinical psychologists could be involved in identifying and recommending the interventions for children and young adolescents with ADHD who have frequent anger problems in school [31]. The school nurses are well placed to be the first health care professional to recognize unhealthy patterns of anger and anger expression. Intervening in unhealthy lifestyles and behaviours early rather than later is important [12]. Early anger management training for children and young adolescents diagnosed as having ADHD may be a promising avenue for preventing more complicated medical and behavioral problems.

## Competing interests

The authors declare that they have no competing interests.

## Authors' contributions

AG conceived the study. AG designed the study. HBH and AG were responsible for gathering of the data. AG drafted the manuscript. AG and HB performed the statistical analysis. All authors contributed to interpreting the results, and read and approved the final manuscript.
